# Dynamics of femtosecond heated warm dense copper with time-resolved L3-edge XANES

**DOI:** 10.1098/rsta.2022.0214

**Published:** 2023-08-21

**Authors:** Ludovic Lecherbourg, Vanina Recoules, Patrick Renaudin, Fabien Dorchies

**Affiliations:** ^1^ CEA, DAM, DIF, Arpajon 91297, France; ^2^ Université Paris-Saclay, CEA, LMCE, Bruyères-le-Châtel 91680, France; ^3^ Université Bordeaux, CNRS, CEA, CELIA, UMR 5107, Talence 33400, France

**Keywords:** warm dense matter, X-ray absorption, laser heating, X-ray sources, Density Functional Theory, femtosecond dynamics

## Abstract

Combining experimental set up and *ab initio* molecular dynamics simulations, we were able to follow the time evolution of the X-ray absorption near edge spectrum (XANES) of a dense copper plasma. This provides a deep insight into femtosecond laser interaction with a metallic copper target. This paper presents a review of the experimental developments we made to reduce the X-ray probe duration, from approximately 10 ps to fs duration with table-top laser systems. Moreover, we present microscopic scale simulations, performed with Density Functional Theory, as well as macroscopic simulations considering the Two-Temperature Model. These tools allow us to get a complete picture of the evolution of the target at a microscopic level, from the heating process to the melting and expansion stages, with a clear view of the physics involved during these processes.

This article is part of the theme issue ‘Dynamic and transient processes in warm dense matter’.

## 1. Introduction

The so-called *warm dense matter* (WDM) regime has been largely studied for two decades [1]. This regime could be found in various domains such as Inertial Confinement Fusion, planetology sciences and astrophysics and in various laser processes. The WDM regime is at the frontier between solid state and plasma physics, where the ions are correlated, and the electrons are degenerated. It is found for densities between 0.1 and 10 times the solid density and temperatures between 0.1 and 10 eV.

One can produce WDM by focusing a short laser pulse at moderate intensity. It creates an out of equilibrium situation for a few picoseconds, where the electrons are hotter than the ions. A number of processes can then be studied, such as, for example, laser deposition and electron thermalization, electron–ion coupling or thermal conduction. The knowledge of these processes is of paramount importance to predict the evolution of larger systems passing through the WDM regime.

We developed a combination of theoretical and experimental tools to study the dynamics of laser heated warm dense copper. Like gold, as a noble metal with a filled electronic d-band below the Fermi level, copper was suspected to exhibit a bond hardening effect when heated up to the WDM regime [2,3]. These studies used *X-ray absorption near edge structure* (XANES) to probe the electronic structure, when copper turns from solid to the WDM regime. XANES spectra present features in a few tens of eV around the L3-edge, which can be correlated with the crystalline structure of the matter.

Other techniques are used to probe WDM, such as reflectivity measurements [4] or X-ray diffraction [5,6]. Reflectivity measurements are indirect measures of the electronic structure, but could have ultra-short time resolutions. X-ray diffraction techniques are powerful tools, which only probed the ionic structure. XANES techniques could overcome these limitations, but time-resolved set-ups are difficult to realize. Few teams used it in the out of equilibrium regime [7,8].

**Figure 1.**
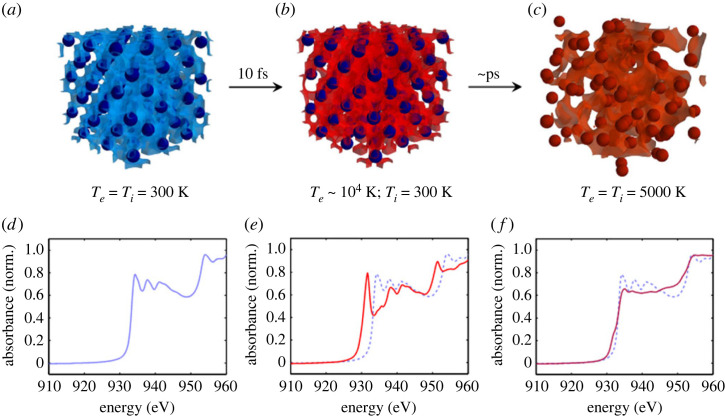
Numerical simulation of the ultrafast non-equilibrium transition of copper from solid to WDM. The energy of a femtosecond optical laser pulse is suddenly deposited in the electrons of the system (femtosecond scale), then progressively transferred to the lattice (picosecond scale). (*a*) Cold solid lattice before heating: the electron temperature $T_{e}$ equals the ion one $T_{i}$. (*b*) Just after heating, a strongly out-of-equilibrium situation is transiently produced where electrons are hot while the lattice is still cold and solid-like. (*c*) A few picoseconds later, the lattice disappears as electrons and ions progress up to their thermal equilibration. (*d*–*f*) Calculated absorption spectra in the XANES region, corresponding, respectively, to (*a*–*c*). The cold XANES signal shown in (*d*) is reported in dashed line in (*e*), (*f*). (Figure taken from [9].)

This paper aims to describe the dynamics of femtosecond heated warm dense copper with time-resolved L3-edge XANES. The principle is sketched in figure 1. It is relevant for thin targets, about a 100 nm thick, and laser fluence of a few $J\;{\mathrm{cm}}^{-2}$. The laser specifically heats the electrons, in a few tens of femtoseconds, followed by energy transport and/or electron–electron thermalization. On a picosecond time scale, melting occurs without significant hydrodynamic expansion. Finally, the electron–ion thermal equilibrium is reached in a few picoseconds, in competition with hydrodynamic expansion that depends on the energy density deposited.

X-ray absorption near edge spectroscopy is used to probe the vacant electronic structure. Such structure strongly depends on the thermodynamic conditions. In order to interpret the spectra, atomic scale simulations are required. The observed dynamics are then compared with hydrodynamic simulations. Such simulations need appropriate microscopic coefficients in the WDM regime, such as the electronic heat capacity, the electronic thermal conductivity and the electron–ion coupling parameter.

The paper is divided into three parts. First, we describe the experimental developments made to improve the time resolution of a laser–plasma X-ray source from picosecond to femtosecond, in order to access to the very beginning of the interaction. Then, we summarize the developments performed at atomic scale with ABINIT code [10], and the code ESTHER used to support hydrodynamic interpretations [11]. The third part is dedicated to the investigation of the time evolution of a thin copper sample. Three experiments are described, which focus on different time scales: the heat process within the first picosecond, the melting about 1 ps and the electron–ion thermal equilibration (a few picoseconds) followed by the hydrodynamic expansion (that can occur over 10 ps).

## 2. Experimental tools: X-ray source

In this section, we describe the X-ray sources developed to study the dynamic of laser heated warm dense copper. The two-temperature phase lasts for picoseconds, but specific properties last for a few picoseconds or less. Efforts have been made to reduce the X-ray source duration from several picoseconds, down to approximately 1 ps by using M-shell thermal emission, and even below 100 fs with betatron radiation.

The ideal properties required for absorption spectroscopy are a high number of photons, and a broadband spectrum. It is often difficult to obtain the desired signal-to-noise ratio in a single shot, then accumulation is mandatory, and so the source stability needs to be high. For L3-edge copper study, a signal-to-noise ratio of at least a few per cent and a spectral resolution of approximately 1 eV are required, within the range of 900–1000 eV.

### (a) M-shell emission

The laser–plasma interaction with a dense target leads to copious X-ray thermal radiation emitted from the hot plasmas. The spectral nature of such an X-ray source depends on the atomic physics, therefore, on the atomic shells and on the target material considered. M-shell emission from high Z plasmas, produced by femtoseconds and picoseconds laser, exhibits bright and broadband spectra, together with a few picoseconds duration [12]. Such a type of X-ray source has been experimentally investigated in the sub-relativistic regime with laser duration varying from approximately 100 fs to approximately 10 ps. Clusters were compared with solid targets, with a particular care to guarantee the closest possible conditions: comparable atomic number Z, atomic shells and photon energy range. Considering the energy of the copper L3-edge, we studied the M-shell emission near 1 keV, from a Xe cluster jet and a CsI solid target. In both cases, a clear difference was observed. In solid, the X-ray emission level monotonically increased with respect to the laser pulse duration, while an optimal duration of a few hundred femtoseconds was evidenced in clusters [13].

**Figure 2.**
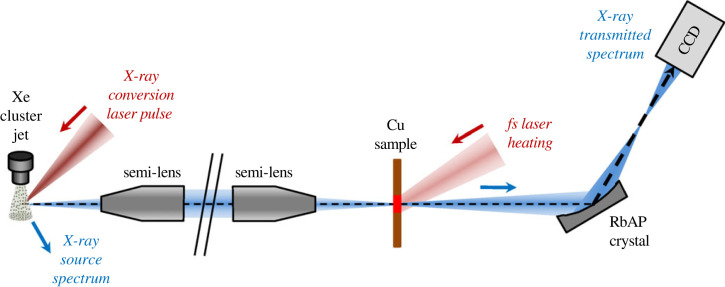
Schematic view of the picosecond time-resolved XANES station developed at CELIA laboratory, based on the M-shell emission. (Figure taken from [13].)

**Figure 3.**
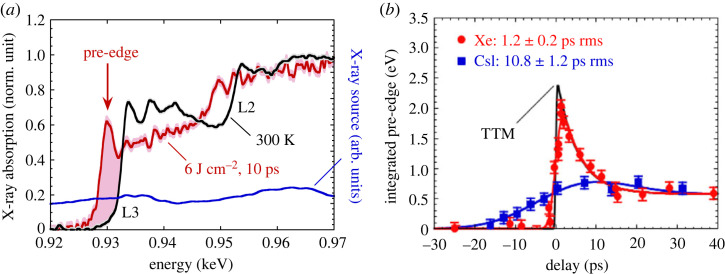
(*a*) Some copper L3-edge XANES spectra registered through a cold sample (black) and a laser heated sample (red). The X-ray source shows broadband spectra in the region of interest. (*b*) Evolution of the pre-edge with the pump-probe delay, when using the M-shell emission from the Xe cluster jet (red), and the CsI solid target (blue), as the X-ray probe. (Figure taken from [13].)

A XANES station has been developed at CELIA laboratory, based on such a M-shell X-ray source [14]. A schematic view is shown in figure 2. The isotropic X-ray emission is collected, transported and concentrated on the copper sample, by the use of polycapillary optics. Two spectrometers are used to register simultaneously the incident X-ray spectrum and the transmitted one through the sample, from which the X-ray absorption spectrum is deduced. Figure 3*a* shows typical XANES spectra at the copper L3-edge. As later explained, the pre-edge feature observed is the direct signature of the electronic temperature $T_{e}$. The evolution of the pre-edge, as a function of the pump (laser) and probe (X-ray) delay, is plotted in figure 3*b*. The prediction from the Two-Temperature Model (TTM) is also plotted for comparison (see further). These measurements indicate a time resolution of 10.8±1.2 ps and 1.2±0.2 ps, respectively, when using solid CsI and Xe cluster, as target for the X-ray source [13].

### (b) Betatron

In order to reach down to a time resolution below 100 fs, few options are available. X-ray free electron lasers (XFEL) are great facilities [15], but have two limitations. The first is the limited access, with only three to four machines worldwide. The second is the very narrow and stochastic spectra. We chose to use the betatron radiation [16], which presents interesting characteristics: very short pulse duration (below 10 fs), broadband spectrum and compatibility with table-top laser systems. Although this source has been known for more than 15 years, it has been too unstable to carry out any realistic XAS study. The recent development improved the stability, which allowed us to design an experimental set-up dedicated to XANES studies [9].

**Figure 4.**
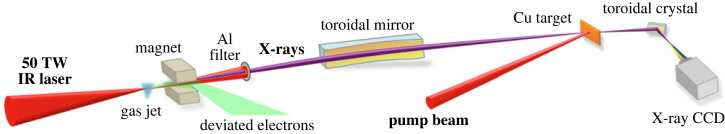
Schematic view of the femtosecond time-resolved XANES experiment at LOA laboratory, using the Betatron X-ray source. (Figure taken from [9].)

Figure 4 presents a typical experimental set-up. A 50 TW laser pulse is focused on a gas jet, which creates an underdense plasma. Electrons from this plasma are accelerated in the laser wakefield. They follow an oscillating trajectory and they emit, in the forward direction, a low-divergence X-ray beam (approx. 10 mrad) with a continuous spectrum extending up to about 10 keV. Typically, $10^{5}$ photons/shot/0.1% BW are emitted near 1 keV. The X-ray radiation is then focused on the copper sample, about 2 m away. This configuration helps to move the spectrometer away from the betatron source, which produces a lot of hot electrons. Finally, the X-rays go into a spectrometer composed of a toroidal-bent crystal and an X-ray camera. This double curvature has two objectives: focus the spectrum in the sagittal plane to increase the number of photon per pixels, and keep a spectral resolution independent of the source size.

**Figure 5.**
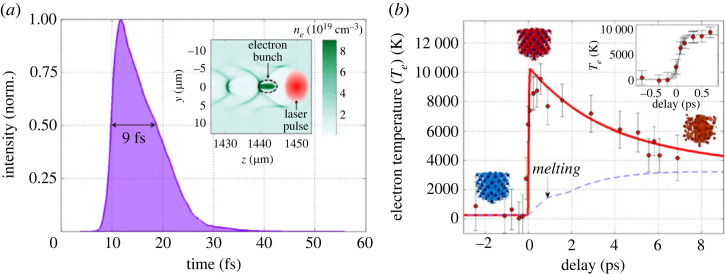
(*a*) Temporal X-ray profile of the Betatron source, resulting from particle-in-cell simulations (inset: corresponding two-dimensional map of the plasma density showing the electron bunch accelerated in the wake of the laser pulse). (*b*) Evolution of the electron temperature deduced from femtosecond time-resolved XANES measurement at the copper L3-edge. (Figure taken from [9].)

Particle in-cell simulation was used to estimate the X-ray pulse duration at 9 fs FWHM (figure 5*a*). With such a pulse, and using the same method as described in the previous section, we were able to measure the electronic temperature dynamic (figure 5*b*). The rise of the electronic temperature was estimated by fitting an error function at 75±25 fs. This time is a combination of three contributions: (i) the pump duration of 30 fs, (ii) the X-ray pulse duration of 9 fs and (iii) a geometric effect of approximately 20 fs between the two extreme parts of the probed diameter (approx. 200 μm). The theoretical time resolution is the quadratic sum of the last two, which gives approximately 40 fs. There are two ways to improve the time resolution: by reducing the X-ray spot size on the sample, or using a geometry where the X-ray probe and the pump are collinear.

## 3. Numerical tools: towards realistic modelling

To fully interpret and understand the experiments, simulations are needed at two levels. The shape evolution of XANES spectra is connected to the electronic structure evolution. This connection is retrieved through Density Functional Theory (DFT)-based molecular dynamics. DFT is also used to compute basic properties needed for hydrodynamic simulations. These hydrodynamic simulations offer a macroscopic view and are used to recover the time evolution of the sample.

### (a) Microscopic simulation

DFT-MD has proven that it is a powerful tool to model optical properties of warm dense plasma, including two temperature systems. Even if X-ray absorption spectra calculation was already computed for solid systems, a methodology for a disordered system has been introduced later. For these systems, one first needs a molecular dynamic simulation to obtain ionic configurations for given thermodynamic conditions. We carry out calculations of the X-ray absorption using DFT-based molecular dynamics simulation (DFT-MD). Then, for selected ionic configurations chosen on the equilibrated part of the trajectory, we perform a more detailed electronic structure calculation. This calculation gives all the required outputs to compute the absorption cross-section. For a given k-point, it is expressed as [17]

3.1 $$\sigma_{k}(\omega )=4\pi^{2}\omega \sum_{n}[1-f(\epsilon_{n,k})]\times |\langle \psi_{n,k}|\nabla |\phi_{\text{core}}\rangle {|}^{2}\times \delta (\epsilon_{\text{core}}-\epsilon_{n}-\hbar \omega ).$$

We employ atomic units, with the electron charge e, Planck’s constant ℏ, the electron mass $m_{e}$ and the fine structure constant α all set to unity. The n summation ranges over the discrete bands (orbitals). $f(\epsilon_{n,k})$ is the Fermi–Dirac occupation factor corresponding to the energy $\epsilon_{n,k}$ of the nth band and for the **k**-point k. The total cross-section is obtained by direct summation over all necessary **k**-points. The core states $|\phi_{\text{core}}\rangle$ are obtained from an all-electron atomic calculation performed during the generation of the PAW dataset using Atompaw code [18]. To take into account the natural linewidth and the resulting spectral broadening of the absorption edges, we use a Lorentzian function to model the delta function with a variable width following [19]. The variable width is necessary as $L_{3}$ and $L_{2}$ are separated by 20 eV. The total absorption is the sum of two contributions: the $L_{3}$-transition from the $2p_{3/2}$ core level to the valence band and the $L_{2}$-transition from the $2p_{1/2}$ to the valence band. At the end, the final spectrum is an average on the previously computed spectra. All details of the calculation can be found in [20].

For transition metals, the effect of spin–orbit coupling (SOC) has to be taken into account. We have implemented the calculation of optical properties for warm dense metals, including SOC for both core and valence orbitals [17]. In this case, Dirac relativistic core wave functions are computed with the Atompaw code to obtain the SOC energy splitting. We obtain a very good agreement with experimental data for both the branching ratio and the spin–orbit splitting. It turns out SOC has little impact on XANES spectra for copper. The valence electronic structure is unchanged and the core states orbitals $\phi_{2p_{1/2}}$ and $\phi_{2p_{3/2}}$ are similar.

**Figure 6.**
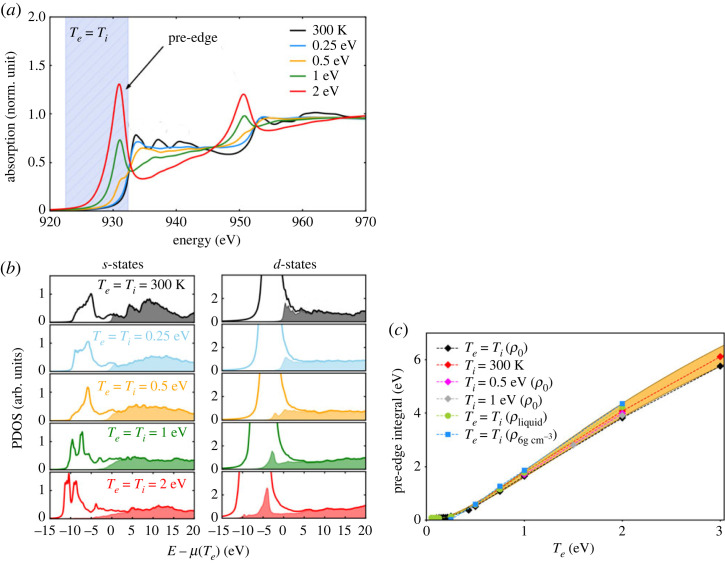
(*a*) Copper XANES spectra calculated for various conditions with $T_{e}=T_{i}$, at solid density. (*b*) s- and d-states for the corresponding spectra. The shade part correspond to the unoccupied states. (*c*) Evolution of the pre-edge integral (shaded part in graph (*a*)) as a function of the electronic temperature $T_{e}$, calculated with different conditions for the ion temperature $T_{i}$ and density ρ. (Figures taken from [20].)

This methodology to compute the absorption cross-section for two-temperature WDM has been validated on a wide variety of systems, and DFT-MD is now a powerful tool to support the interpretation of measured X-ray absorption spectra for WDM (e.g. [7,8,21]). For aluminum, it has been shown that the electronic temperature can be read directly on the slope of K-edge spectra [22]. The modification induced on the spectra by temperature and density depends on the material and we cannot use the same criteria for copper. Each material needs a dedicated study. In the case of copper, the shape is modified in two ways. It is summarized in figure 6*a*,*b*. First, as copper is a noble metal with almost filled *3d* states at ambient conditions, they do not appear on the XANES spectrum near the L-edge at ambient condition (black curves). But, when the temperature of the electrons increases (coloured curves), the *3d* states are partially depopulated, leading to a pre-edge on the absorption spectrum and a reduction of the absorption behind the edge. The amplitude and shape of the pre-edge is directly linked to the modification of 3d electronic population. The spectral integral of the pre-edge can be used to deduce the electronic temperature for a wide range of temperatures and densities, as illustrated in figure 6*b*. This leads to a useful tool to associate an electronic temperature to experimental XANES spectra.

Second, the modulations above the edge disappear when the temperature increases as solid copper turns to a liquid state when local order disappears. This signature of the loss of order has already been observed for Al, Mo and Fe. In the case of copper, this has been linked to the van Hove singularities vanishing [23].

Once validated on calculated spectra, this methodology is used directly on experimental spectra to retrieve the time evolution of the electronic temperature in femtosecond laser heated copper and to give a time scale for the loss of local order.

### (b) Macroscopic simulations

At a macroscopic level, the dynamics between electrons and ions could be described by a set of two differential equations, called the TTM [24]:

$$C_{e}\partial_{t}T_{e}=-G_{ei}(T_{e}-T_{i})+\partial_{x}(\kappa_{e}\partial_{x}T_{e})+S$$

and

$$C_{i}\partial_{t}T_{i}=G_{ei}(T_{e}-T_{i})+\partial_{x}(\kappa_{i}\partial_{x}T_{i}),$$

where $C_{e/i}$ are the heat capacities, $\kappa_{e/i}$ are the heat conductions and $G_{ei}$ is the electron–ion coupling parameter. The electronic and ionic temperatures, $T_{e/i}$, and the source term, S, are functions dependent on space and time. This model is then coupled with a hydrodynamic expansion governed by an equation of state. This combination gives the thermodynamic evolution of the target, such as the electronic and ionic temperatures, or the density.

The physics lies in the description of these coefficients. Their validity needs to be questioned, in particular in the WDM regime. One common option is to interpolate from the solid state to the plasma physics. Unfortunately, it often lacks effects due to the complex structure of WDM. Studies have then realized to calculate these coefficients from atomistic simulations [25], and recent developments are in progress [26].

**Figure 7.**
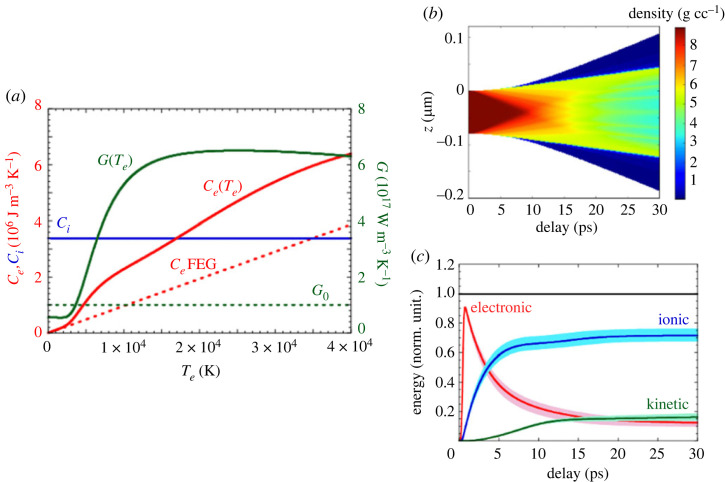
Results of two-temperature hydrodynamic calculations performed when $0.4\;J\;{\mathrm{cm}}^{-2}$ is uniformly deposited in an 80 nm copper foil. (*a*) $T_{e}$-dependent coefficients G and $C_{e}$ used in the simulation, as derived from [25]. (*b*) Two-dimensional (longitudinal/temporal) evolution of the density. (*c*) Corresponding time evolution of the energy balance. (Figure taken from [27].)

Figure 7*a* shows two major differences with $C_{e}$ and $G_{ei}$ coefficients. When calculated with atomistic simulations, $C_{e}$ shows that it could be greater by a factor of 2 than the free electron gas model, while $G_{ei}$ strongly depends on the electronic temperature. For the sake of completeness, $C_{i}$ is also added in the figure, and it does not depend on the electronic temperature. Figure 7*b* shows the time evolution of the density of a thin foil calculated with the hydrodynamic code ESTHER [11]. It shows the rarefaction wave inside the sample, which lasts approximately 10 ps, and a small part of the sample expanding at very low density. Figure 7*c* shows the total energy in the electrons, ions and also the ions kinetic energy from the previous simulations. We clearly see the rapid drop and rapid increase of electronic and ionic energies below 5–10 ps, but also that about 15% of the energy goes into the ion kinetic for times above 10–15 ps, where the thermal expansion plays a significant role.

In laser–matter interaction, the energy is given to the electrons. The total absorbed energy, represented by the S term in the TTM equations, is difficult to calculate. It strongly depends on the model used to calculate the dielectric index. To overcome this difficulty, experimental data could be performed to measure the reflected and transmitted beam, in order to determine the total absorbed energy. Such experiments are not technically difficult, but careful attention to the calibration of the various elements is mandatory to obtain significant data [27].

## 4. Time evolution of a femtosecond laser heated copper foil

### (a) Heating, during the first picosecond

At the atomic scale, an 800 nm (1.55 eV) pump pulse excites the electrons from the 4s-band, in the skin depth of a copper sample. Chen *et al.* [28] have shown a flux threshold in gold, which separates how the electronic energy is transported. At lower flux, the sample is heated uniformly, while at higher flux an inhomogeneity is measured. These observations have been explained with two types of energy transport. At low flux, the excited electrons are transported ballistically over approximately 100 nm [29]. At higher flux, the electrons are excited to higher energy, thermalized very quickly (femtosecond time scale [30]) and followed by diffusive transport.

**Figure 8.**
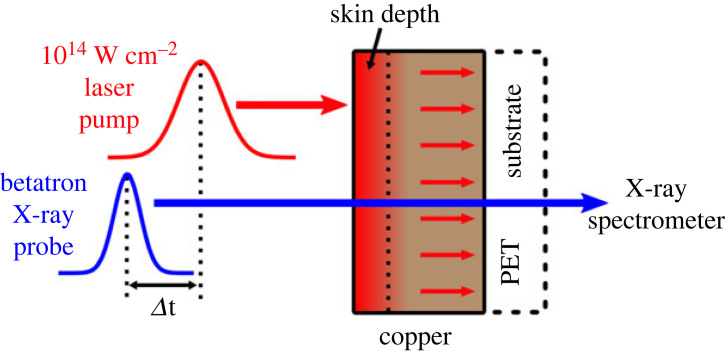
Schematic view of the laser pump/X-ray probe experiment. The laser, with an intensity of $10^{14}\;W\;{\mathrm{cm}}^{-2}$, heats a copper sample of 100 nm thickness deposited on a PET substrate. (Figure taken from [31].)

**Figure 9.**
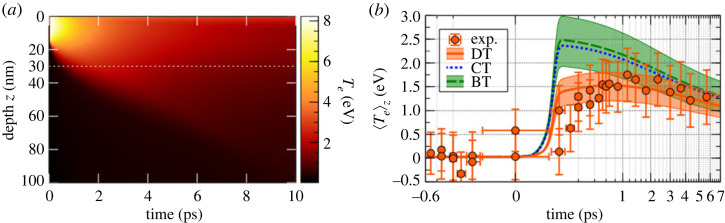
(*a*) Evolution of the electronic temperature as a function of the initial depth and time, for diffusive transport. The laser deposition happens in the first 20 nm. (*b*) Electronic temperature dynamics retrieved from XANES spectra, compared with three TTM simulations: ballistic transport (BT), diffusive transport (DT) and composite transport (CT). (Figure taken from [31].)

We performed time-resolved XANES on a copper sample, with an approximately 40 fs resolution, in the diffusive regime [31]. It allowed us to probe the electronic temperature below the first picosecond. Figure 8 shows a scheme of the set-up: an 800 nm pump beam, with an intensity of $1\times 10^{14}\;W\;{\mathrm{cm}}^{-2}$, heats a copper sample of 100 nm thickness. The heat propagates through the sample within 10 picoseconds. With a controlled delay, the sample is probed with betatron radiation and XANES spectra are recorded. Using the technique previously described, we retrieved the electronic temperature from the integral of the pre-edge structure, and we were able to plot electronic temperature as a function of time (figure 9*b*).

To analyse such dynamics, we performed hydrodynamic simulations with three different heat processes: (i) first, we considered a homogeneous heating over the whole sample to simulate a ballistic transport; (ii) then we considered an inhomogeneous heating in the first 20 nm of the sample, to simulate a diffuse transport (figure 9*a*); and (iii) finally, we considered a mixture of both transport, i.e. 60% of ballistic transport plus 40% of diffusive transport. This last simulation was performed to simulate a saturation effect of the ballistic transport. Indeed, Chen *et al.* proposed that the evacuated flux by ballistic electrons is limited by the energy they can transport: $Q_{NT}=n_{e}E_{F}v_{F}$, where $n_{e}$, $E_{F}$ and $v_{F}$ are the electron density, the Fermi energy and Fermi velocity, respectively. In our case, $Q_{NT}$ is 60% of the absorbed flux. The simulations show clearly that only the diffusive transport reproduces the electronic temperature measured during the first picosecond.

We showed that for an energy range above the flux limit, the energy transport is governed by a diffusive regime rather than a ballistic regime. Femtosecond time-resolved XANES allows us to constrain the nature of electron–electron collisions during the heating phase. This study focused on the energy transport during the first picosecond, but other physical properties can also be studied at a longer time scale.

### (b) Melting (approx. 1 ps)

**Figure 10.**
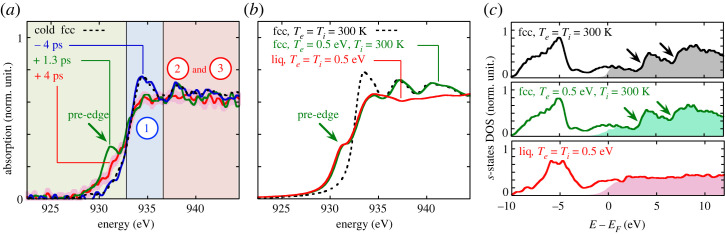
(*a*) Some time-resolved XANES spectra measured at $F_{\text{abs}}=0.065\;J\;{\mathrm{cm}}^{-2}$. (*b*) Some calculated XANES spectra. (*c*) Corresponding computed projected DOS on *s*-states. The van Hove singularities characteristic of the fcc crystalline phase are indicated with arrows. (Figure taken from [23].)

Depending on the considered material and absorption edge, XANES spectroscopy can provide information about the crystalline structure. This is particularly the case for the L3-edge of copper, as we demonstrated in [20,23]. Some features, just above the edge (labelled 2 and 3 in figure 10*a*), are associated with van Hove singularities in the electron structure (indicated with arrows in figure 10*c*). Such singularities are specific to the electronic band structure, therefore, to the crystalline structure (here fcc). When the solid copper turns to liquid, the lattice periodicity is lost and the consequent XANES post-edge features vanish.

This was exploited to investigate the ultrafast melting dynamics at the subpicosecond time scale, in the range of specific energy density from 1 to $5\;\mathrm{MJ}\;{\mathrm{kg}}^{-1}$. A simultaneous control of the electron temperature dynamics is provided by the pre-edge feature in the same X-ray absorption spectra. Since the observation of the post-edge features is more demanding in terms of signal-to-noise ratio, we have used the M-shell X-ray source presented in §2a, which provides more X-ray photons on the detector per shot. The price to pay is the limited time resolution of 1.2±0.2 ps. However, this was sufficient to observe clear out-of-equilibrium situations, as plotted in green in figure 10*a* (registered with a delay of 1.3 ps after the laser heating). The pre-edge reveals a high electronic temperature (approx. 0.5 eV, well above the expected melting temperature, approx. 0.1 eV), while the post-edge features demonstrate a still solid crystalline structure.

**Figure 11.**
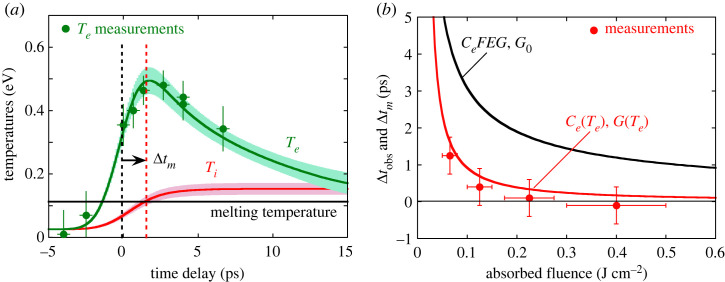
(*a*) Time evolution of electron ($T_{e}$) and ion ($T_{i}$) temperatures estimated with the two-temperature code, compared with $T_{e}$ measurements at $F_{\text{abs}}=0.065$ $J\;{\mathrm{cm}}^{-2}$ (circles). (*b*) Time delay $\Delta t_{\text{obs}}$ measured for the electron structure transition from fcc to liquid, compared with calculated melting time $\Delta t_{m}$, as a function of the absorbed laser fluence. (Figure taken from [23].)

The time $\Delta t_{\text{obs}}$ needed for the crystalline order to disappear is inferred from the time-resolved measurements. The temporal behaviour of the electron temperature is plotted in figure 11*a*. It fits very well with the prediction of two-temperature macroscopic calculations. The corresponding calculated dynamics of the ion temperature $T_{i}$ is also plotted (in red). The thermal melting time $\Delta t_{m}$ is estimated when $T_{i}$ exceeds the melting temperature. Figure 11*b* shows a good agreement between measurements ($\Delta t_{\text{obs}}$) and calculations ($\Delta t_{m}$), and this in a wide range of absorbed fluence (up to $0.4\;\text{J}\;{\text{cm}}^{-2}$).

The melting time is observed near approximately 1 ps at the lowest fluence investigated ($0.065\;\text{J}\;{\text{cm}}^{-2}$), and it decreases when the energy deposition increases. The overall observations are fairly well reproduced by the simple TTM, provided that $T_{e}$-dependent coefficients are considered [25] and assuming that the melting occurs when the ion temperature exceeds the melting temperature. Data do not indicate any bond hardening effect, which is not surprising since this effect has been predicted for $T_{e}\geq 3$ eV [3], while $T_{e}$ does not exceed 2 eV in the present work.

### (c) Electron–ion thermal equilibration and hydrodynamic expansion (approx. 1–10 ps)

As seen just above (for example, in figure 11*a*), the fast laser energy deposition in electrons is followed by energy transfer from electrons to ions on a few picosecond time scales. This phenomenon is understood as the electron–ion thermal equilibration, which is described in the TTM with the use of a set of thermodynamic coefficients, mainly the electron–ion coupling $G_{ei}$, but also electron and ion heat capacities $C_{e/i}$. We used $T_{e}$-dependent coefficients from Lin *et al.* [25], keeping in mind that they have been evaluated assuming a solid-state density of states (DOS) for both electrons and phonons.

In addition, the fast energy laser deposition in a thin film induces high pressure, which can drive significant hydrodynamic expansion. In the sections above, this phenomenon could be ignored most of the time, thanks to the relatively low absorbed energy flux (considered for the melting dynamic study) and the ultra-short time scales (femtosecond scale considered for the heating transport study). But it may have a significant impact on the energy balance, even at moderate energy flux. It should even become a severe issue when investigating higher energy flux, even at the picosecond or the femtosecond scales.

This is illustrated in the macroscopic simulations reported in figure 7, and performed with an absorbed fluence of $0.4\;\text{J}\;{\text{cm}}^{-2}$. In this situation, well above the melting threshold, the electron temperature exceeds 2 eV just after the laser heating. Both sample sides start to expand from the beginning of the interaction. The frontiers between solid and liquid densities propagate through the copper foil at the sound velocity, and reach the centre of the foil in about 10 ps, which is only a little longer than the electron–ion thermal equilibration. The right part of the figure shows the evolution of the energy balance that illustrates the different time scales. The energy transfer from electrons to ions (thermal energies) occurs over a few picoseconds, but the electron energy continues to be transferred to the hydrodynamic expansion. At the end of the process, about 15% of the energy is converted into kinetic energy.

**Figure 12.**
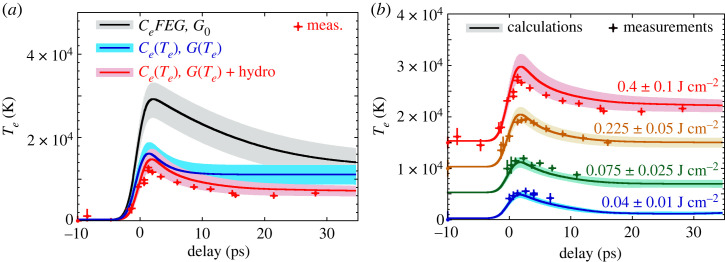
Time evolution of $T_{e}$ retrieved from time-resolved XANES measurements. (*a*) Data registered with an absorbed fluence of $0.4\;J\;{\mathrm{cm}}^{-2}$, and compared with different calculations. (*b*) Data at different laser fluences compared with the hydrodynamic simulations performed with $T_{e}$-dependent coefficients. (Figure taken from [27].)

Taking advantage of the $T_{e}$ diagnostic provided by the pre-edge near the copper L3-edge, we performed several time-resolved experiments with the XANES station presented in figure 2. Some data are presented in figure 12*a* (red crosses). Comparison with macroscopic simulations performed with different sets of TTM coefficients demonstrates that $T_{e}$-dependent coefficients are required to reproduce the observed maximum value of $T_{e}$, as well as the hydrodynamic expansion to get the best fit at longer delays [27].

Measurements have been performed for different incident fluences. Results are plotted in figure 12*b*. A very good overall agreement is observed with simulations, both in amplitude and in the equilibration dynamics. No free parameter is introduced in the calculations as the absorbed fluences are set to measured values. For the lowest fluence ($0.04\;\text{J}\;{\text{cm}}^{-2}$ absorbed), the impact of the hydrodynamic expansion in the calculated $T_{e}(t)$ goes below the experimental error bars.

This study demonstrates that the picosecond dynamics of the electron–ion thermal equilibration is well understood. Even if the investigated conditions are far from the solid-state assumptions of Lin *et al.* [25], a satisfactory agreement is found with the temperature-dependent electron–ion coupling factor that they proposed. In the highest temperature range investigated (up to approx. 2 eV), the hydrodynamic expansion induced by the high pressure achieved cannot be ignored and affects significantly the energy balance between electrons and ions.

## 5. Conclusion

We have developed experimental and theoretical tools to study the dynamic of out-of-equilibrium WDM. We used the XANES technique to probe the electronic states of the copper sample near the L3-edge. XANES spectra are compared with microscopic simulations (DFT-MD with ABINIT) and the dynamics is compared with macroscopic simulations (TTM with ESTHER code).

In the first picosecond, we investigated electronic heating processes. We have demonstrated that the energy transport is modified above a flux limit, from a ballistic regime to a diffusive regime. Then, at a longer time scale (approx. 1 ps), we observed the melting time to be very fast, and well reproduced by TTM simulations. The melting was retrieved from XANES spectra by following the evolution of van Hove singularities. Finally, at a time scale over a few tens of a picosecond, we followed the temporal dynamics for various energy deposition. We showed that the TTM reproduced the experiment if the macroscopic coefficients were well adapted in the WDM regime, i.e. calculated from DFT-MD microscopic simulations. Moreover, at high energy deposition, the thermal expansion plays a significant role.

In the domain experimentally investigated, we showed that TTM simulations describe accurately the observations, from the energy transport, the melting time and the hydrodynamic expansion and cooling. These simulations required to have the correct coefficients, calculated from DFT-MD simulations. In order to explore bond hardening effects, it is necessary to reach higher WDM temperatures. This requires to improve further the signal-to-noise ratio of the femtosecond XANES beam line. To go beyond the TTM model, particularly at the very beginning of the heating, non-thermal effects could be explored by pumping the medium into a lower energy band, from the 3d-band with a 400-nm wavelength beam or from the inner shell using X-ray beams such as XFEL. This methodology could be applied to other materials with different electronic structures, such as transition metals or dielectric. However, it is mandatory to perform DFT-MD simulations in order to interpret XANES features.

## Data Availability

This article has no additional data.
